# PROSPERO’s progress and activities 2012/13

**DOI:** 10.1186/2046-4053-2-111

**Published:** 2013-12-11

**Authors:** Alison Booth

**Affiliations:** 1 Centre for Reviews and Dissemination, University of York, York YO10 5DD, UK

**Keywords:** NIHR, PROSPERO, Protocol, Register, Systematic reviews, Transparency, Update

## Abstract

PROSPERO is an international database of prospectively registered systematic reviews in health and social care. Between July 2012 and June 2013, 1,106 registrations were added, bringing the total since launch in February 2011 to 1,704. The value of the growing number of records is reflected in a 117% increase in page views in the first half of 2013 compared with the first half of 2012. Developments over the year included expansion of scope, improvement of the registration form and easier access to information on how to register.

## Letter

### Background

PROSPERO was launched in February 2011 as the first open access international prospective register for systematic review protocols. The aim of the register is to help prevent unintended duplication of systematic reviews by allowing those commissioning or planning reviews to identify whether there are any relevant reviews already underway. PROSPERO also aims to provide transparency in the review process and help identify potential biases by enabling comparison of reported review findings with what was planned in the protocol. Registration, which is free, requires entry of a minimum dataset that includes key information about the systematic review design and brief administrative details and was developed and agreed through international consultation. This letter briefly outlines activity and progress of PROSPERO from 1 July 2012 to 30 June 2013.

### Progress

**Figure 1:**
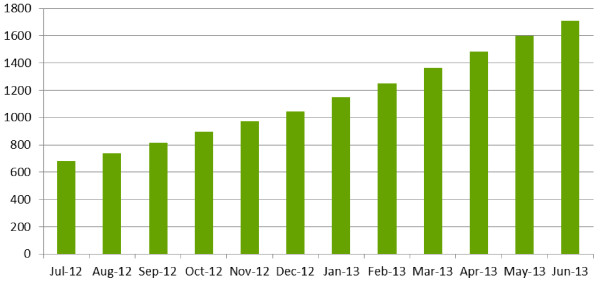
Cumulative totals for new registrations on PROSPERO.

**Figure 2:**
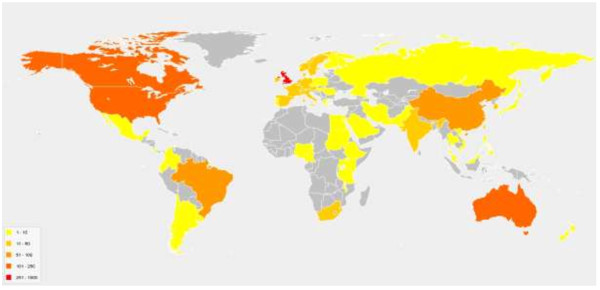
**Countries where registered reviews are being undertaken.** (From launch Feb 2011 to 30 June 2013). Registrations are received from all round the world, with 62 different countries involved. New additions to the 2012/13 period were Portugal, Iran, Colombia, Czech Republic, Ethiopia, Malta, Nigeria, Pakistan, Qatar, Romania, Saudi Arabia, Costa Rica, Hungary, Russia, Lebanon and Fiji.

Since its inception, there is evidence that PROSPERO is gaining increasing acceptance and visibility. Numbers of protocol registrations and register users have increased rapidly; 1,106 records were added to PROSPERO between July 2012 and June 2013, bringing the total number of registrations to 1,704 (cumulative totals are shown in Figure 1). Additionally, 175 protocols were submitted but not accepted, usually because the review had progressed too far for inclusion. As PROSPERO is a prospective register, reviews should ideally be registered before screening against eligibility criteria commences. The majority of registrations came from England (471), primarily because the National Institute for Health Research (NIHR) have introduced mandatory registration for all eligible reviews they fund. Canada (191), the USA (158), Australia (143) and Brazil (86) also had high registration numbers. Worldwide registration statistics are shown in Figure 2.

PROSPERO usage statistics showed that 1,134,605 pages were viewed by 39,808 unique client internet providers (IPs) over the past year. As IP addresses can represent either a single user or a whole organisation (for example, the National Health Service in England), we know that this is a conservative estimate of usage. Nevertheless, register usage greatly increased in 2013 with 117% more page views in the first half of 2013 compared with the same period in 2012. This reflected the growing number of searchable records in the register.

In January 2013, we published data on the utility of PROSPERO in its first year [1]. One outcome from this review was an expansion in scope. PROSPERO now accepts systematic reviews of reviews and systematic reviews of methodological issues, provided they contain at least one outcome of direct patient or clinical relevance. The registration form was also improved; for example, users can now save draft versions of their protocol as pdf files or word processing documents to share with colleagues prior to submission. We also improved access to information on how to register; for example, the inclusion criteria are now available directly from the home page.

Presentations about PROSPERO were well received at the EQUATOR Scientific Symposium in Freiburg [2] and the 2nd International Symposium on the Systematic Reviews in Laboratory Animal Science in Edinburgh [3]. Although reviews of animal studies are not eligible for inclusion in PROSPERO, it is hoped that a sibling register could be launched to support work in this area. New supporters of PROSPERO as a freely accessible prospective register of protocols include BioMed Central (open access publisher of over 250 peer reviewed journals) and the Head and Neck Optical Diagnostics Society. The Canadian Institutes for Health Research, who were supporters from the very beginning, also produced a new statement commending PROSPERO as a means of increasing transparency in research, which ultimately helps to improve health outcomes.

### Plans for the future

Plans for further PROSPERO developments include implementation of a new administration system with such functions as automated email reminders to help registrants keep their records up to date. The system will also facilitate the efficient addition of new Cochrane protocols to the register. A new search interface for the website is currently under development and is being informed by feedback from a recent survey of PROSPERO registrants and users.

The PROSPERO team would like to thank the advisory group for their continued advice and support, and all registrants and register users. We always welcome feedback and would like to hear more from users, particularly if and how the register has been useful.

## Abbreviations

CRD: Centre for reviews and dissemination; IP: Internet provider; NIHR: National Institute for Health Research.

## Competing interests

The development and ongoing management of PROSPERO is supported by the Centre for Reviews and Dissemination’s (CRD) core work programme, which is funded by the National Institute for Health Research, England; the Department of Health, Public Health Agency, Northern Ireland and the National Institute for Social Care and Health Research, Wales.

AB is a research fellow at NIHR CRD, University of York, UK, and is responsible for the development and maintenance of PROSPERO.
